# Are socio-economic inequalities in breast cancer survival explained by peri-diagnostic factors?

**DOI:** 10.1186/s12885-021-08087-x

**Published:** 2021-05-01

**Authors:** Laura M. Woods, Bernard Rachet, Melanie Morris, Krishnan Bhaskaran, Michel P. Coleman

**Affiliations:** 1 Department of Non-Communicable Disease Epidemiology, Faculty of Epidemiology and Population Health, London School of Hygiene & Tropical Medicine, Keppel St, London, WC1E 7HT UK; 2 Department of Health Services Research and Policy, Faculty of Public Health and Policy London School of Hygiene & Tropical Medicine, 15-17 Tavistock Place, London, WC1H 9SH UK

**Keywords:** Breast neoplasms, Comorbidity, Early diagnosis, Socioeconomic factors, Survival analysis, Primary health care, Peri-diagnostic period

## Abstract

**Background:**

Patients living in more deprived localities have lower cancer survival in England, but the role of individual health status at diagnosis and the utilisation of primary health care in explaining these differentials has not been widely considered. We set out to evaluate whether pre-existing individual health status at diagnosis and primary care consultation history (peri-diagnostic factors) could explain socio-economic differentials in survival amongst women diagnosed with breast cancer.

**Methods:**

We conducted a retrospective cohort study of women aged 15–99 years diagnosed in England using linked routine data. Ecologically-derived measures of income deprivation were combined with individually-linked data from the English National Cancer Registry, Clinical Practice Research Datalink (CPRD) and Hospital Episodes Statistics (HES) databases. Smoking status, alcohol consumption, BMI, comorbidity, and consultation histories were derived for all patients. Time to breast surgery was derived for women diagnosed after 2005. We estimated net survival and modelled the excess hazard ratio of breast cancer death using flexible parametric models. We accounted for missing data using multiple imputation.

**Results:**

Net survival was lower amongst more deprived women, with a single unit increase in deprivation quintile inferring a 4.4% (95% CI 1.4–8.8) increase in excess mortality. Peri-diagnostic co-variables varied by deprivation but did not explain the differentials in multivariable analyses.

**Conclusions:**

These data show that socio-economic inequalities in survival cannot be explained by consultation history or by pre-existing individual health status, as measured in primary care. Differentials in the effectiveness of treatment, beyond those measuring the inclusion of breast surgery and the timing of surgery, should be considered as part of the wider effort to reduce inequalities in premature mortality.

## Background

Patients living in more deprived localities have lower cancer survival in England [1–4]. The avoidable mortality associated with these socio-economic differences is considerable [5]. There are three potential routes by which these inequalities might arise [6, 7]: tumour factors (more aggressive disease, more advanced disease arising from differential ease of access and availability of appointments, and, or screening), patient factors (differential pre-existing comorbidities, health or nutritional status, leading to less effective or under-treatment), and health system factors (differential referral patterns from primary care, or differential treatment within secondary care).

To date, the relative contribution of these mechanisms in explaining the persistence of socio-economic differences in England has focussed on a variety of factors. These include the examination of patterns of survival by screening status [8–10], analyses of routine data from secondary care [11–16] and the equalisation of treatment [17–19]. The presence of factors measured in primary health care, such as the presence of other diseases, obesity, smoking history, alcohol consumption, as well as the total number of consultations attended by the patient may also be associated with these inequalities. However, their role in explaining survival differentials has not been considered outside our own analysis of screening-eligible women diagnosed with breast cancer [20, 21].

In this study, we specifically consider the relative impact of a) pre-existing individual health status (comorbidity and detrimental health behaviours) together with b) primary care consultation history upon socio-economic patterns in breast cancer survival, using linked routine cancer registration and primary care data. These factors represent potentially modifiable factors which could help to reduce inequalities and avoidable mortality for women with breast cancer as well as for patients diagnosed with other socio-economically patterned diseases.

## Methods

### Data sources

The English National Cancer Registry (CR) was individually linked to Clinical Practice Research Datalink ‘GOLD’ (CPRD) which contains data contributed by practices using Vision® software [22] and Hospital Episodes Statistics (HES) databases. The CR-CPRD linkage took place on two different occasions: in 2010 for diagnoses 1988–2004 and in 2016 for diagnoses 2005–2010. Hospital Episodes Statistics (HES) data were available for the later period only.

### Deprivation

We used ecologically-derived measures of income deprivation for each woman: quintiles of the 1991 census-based Carstairs index [23] for women diagnosed 1988–1995, and the English Indices of Multiple Deprivation (IMD) income domain from 1998 onwards [24]. Although each of these scores use slightly different underlying variables, they both aim to quantify relative deprivation by computing a score from the socio-economic characteristics of very small areas using the census or routinely collected administrative data (Carstairs: car ownership, overcrowding, social class and unemployment, IMD: receipt of various means-tested benefits). The areas used for each score are those defined at the UK’s decennial census (EDs in 1991 c.500 persons; LSOAs in 2001 and 2011 c.1500 persons but designed to be as socially homogenous as possible) and are the smallest administrative geography available at any given time point. Deprivation categories were derived from the score temporally closest to each woman’s date of diagnosis on the basis of her residential address.

### Co-variables

We used information from the cancer registry to derive each woman’s date and age at diagnosis, tumour characteristics and date of death (if applicable). We derived stage of disease at diagnosis using all relevant available clinical information [25]. Each women’s individual smoking status (non- or ex-smoker, current smoker), alcohol consumption status (non-, ex-, current drinker) and body mass index [26] were extracted from CPRD records as previously described [20]. The Charlson comorbidity score [27] was derived from data recorded in the 18-month period between 2 years to 6 months before diagnosis [28] using information from both CPRD and HES data for patients diagnosed after 2005. The total number, as well as the number of “breast-related” vs. “not breast-related” consultations along with the number of referrals for breast cancer were derived for 18-month period immediately prior to diagnosis. Breast-related symptoms included any mention of separate breast symptoms, within the same consultation or reported at different times, including breast lump, breast pain, skin changes, discharging bleeding or inverted nipples. We adopted the conservative approach of considering only consultations with a doctor (GP), excluding CPRD records relating to nurse or other practitioner appointments, as well as all administrative events such as telephone calls, letters, or the issuing of repeat prescriptions. This avoided potentially recording one symptom more than once, or inflating a woman’s total number of consultations by the inclusion of non-clinical events. Time in days from the last breast-related consultation to diagnosis (as an indication of time elapsed from referral to diagnosis) was calculated for all patients and from diagnosis to first major breast cancer surgery (within 18 months, defined using OPCS-4 codes, the classification used by clinical coders within National Health Service) for women diagnosed after 2005. A specific category for missing data was available for stage, and we similarly coded women as ‘missing’ if no information on smoking, alcohol and BMI could be obtained. It was not possible to distinguish the difference between ‘none observed’ and ‘missing information’ for pre-existing comorbidities, symptoms, referrals or surgery. For these variables, ‘none recorded’ was assumed to equate to the non-observation of the relevant factor in primary and secondary care. Multinomial regression (categorical variables) and non-parametric tests for trend (continuous variables) [29] were used to assess the differences between deprivation categories.

### Net survival estimation

Net survival is the survival probability the patients would experience if their only possible cause of death were breast cancer. It is independent from other causes of death (expected mortality, which varies in particular by age and deprivation) and reflects the prognosis of the disease. We estimated net survival by each co-variable using the non-parametric Pohar-Perme estimator [30, 31] implemented in *stns* [32]: software available for Stata 16 [33]. This is the most widely used, unbiased estimator of net survival. Controlling for expected mortality (or its counterpart, expected survival) required the use of information from deprivation-specific life tables for the general population of England [34]. Survival estimates were derived for all co-variables for the data as a whole as well as by time period (1988–1998, 1999–2004 and 2005–2010).

### Multivariable excess hazard modelling

We fitted flexible parametric excess hazard regression models using *stcrs* [35] in order to estimate the excess hazard ratio of death (i.e. death related to breast cancer) within the first 5 years following diagnosis. This approach models the excess hazard on the log-hazard scale, reducing computational intensity, and also allows the estimation of both time-dependent and non-linear covariable effects. We examined the mechanism giving rise to missing values for the four variables within the dataset with incomplete data (stage, smoking, alcohol consumption and BMI) using logistic regression. In order to account for the impact of these missing data in the analysis, we implemented a five-fold multiple imputation which was enough to obtain stable estimates and variance. Imputation models were fitted separately for each deprivation quintile to enable interactions to be considered, and included all variables of interest. Missing values for BMI were derived from a linear regression model, stage from an ordered logistic model and smoking and alcohol from multinomial regression. Estimates were recombined using Rubin’s rules [36]. Initial excess hazard models included, a priori, age, year of diagnosis, deprivation and stage of disease at diagnosis. We tested for non-linearity of each of these variables using restricted cubic splines with 3 degrees of freedom (2 internal knots) for age and year, and the ordered categorical form of the variable for deprivation using the Stata sub-command *mi test* [36] (*p*-value < 0.05). Peri-diagnostic variables which were observed to have a significant association with both deprivation and net survival in the univariable analyses were included in turn, first those relating individual health status, then individual consultation history in primary care. Models used all disease stages, then were subsequently fitted only to TNM stage I or II. Models were derived by follow-up time in order to assess time-variance. Finally, we repeated all analyses restricting the cohort to diagnoses 2005–2010. We used precisely the same strategy, but included in the model the number of days from diagnosis to major breast surgery and the Charlson comorbidity score derived from both CPRD and HES.

## Results

### Cohort & data linkage

**Fig. 1:**
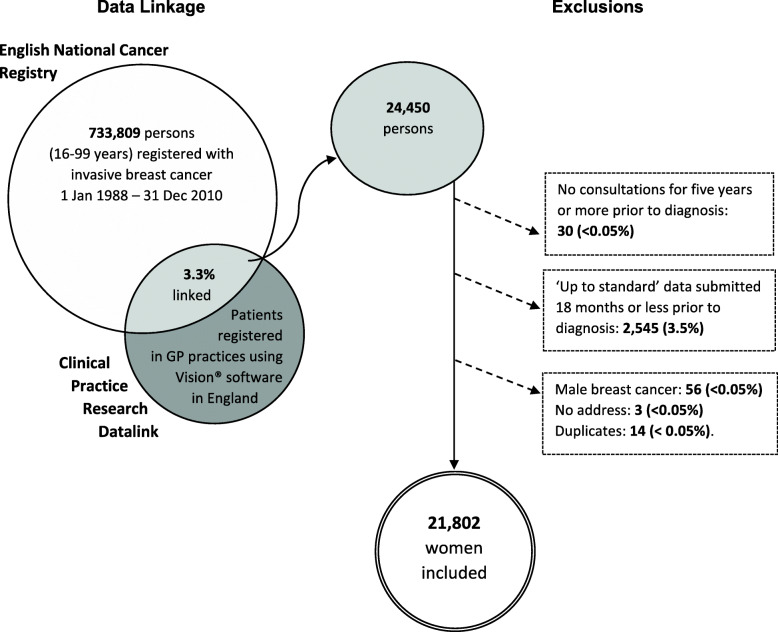
Schematic displaying numbers of persons registered in each database, data linkage proportion, numbers and percentages of eligible persons excluded

Out of the 733,809 persons aged 16–99 years in England recorded in the National Cancer Registry as having being diagnosed with invasive breast cancer between 1 January 1988 and 31 December 2010, we analysed 21,802 women for whom follow-up was complete up to 31 December 2014 (Fig. 1).

### Descriptive analyses

A third of the women died on or before the end of follow-up (Table 1). Women living in deprived areas were on average 2 years older at diagnosis and less likely to be diagnosed in the screening age range 50–69 (*p*-value < 0.001). They were less frequently diagnosed with localised (Stage I) disease (3.3% difference. 95% CI 1.4–5.2) and much more likely to die during the study period (11.1% difference, 95% CI 8.8–13.5). They were also more likely to be recorded as current or ex-smokers (13.9% difference, 95% CI 11.6–16.3), non- or ex-drinkers (15.7% difference 95% CI 13.6–17.8), and have a recorded BMI above 24 (11.6% difference 95% CI 14.1–9.3). There was a very strong linear association with pre-existing co-morbidities and deprivation, with 82.2% of women living in the least deprived areas having no pre-existing condition compared to 70.7% of women living in the most deprived areas (difference 11.4 95%CI 9.6–13.5). Women in deprived areas had a higher mean number of consultations overall (9.6 vs 8.5, *p*-value < 0.001), but a slightly lower number of breast-related consultations compared with women living in more affluent areas (0.4 fewer, 95% CI 0.1–0.8). Women living in the most deprived areas reported a similar number of breast symptoms to the GP prior to diagnosis than women living in the most affluent areas (53.9% vs 53.1% reporting at least 1). However, women living in middle- to deprived areas (quintiles 3 and 4) reported fewer (*p*-value < 0.01). The average time from symptom report to diagnosis was longest amongst women in the most affluent two quintiles (32.7 days) but not notably shorter in any other group (30.7–32.4 days). These overall patterns were similar in the data set restricted to diagnoses after 1 January 2005 (data not shown).

**Table 1:** Numbers and percentages by deprivation and co-variable status: (A) women diagnosed 1988–2010 and (B) 2005–2010

	Total		1 - Least deprived		2		3		4		5 - Most deprived		***p***-value^**2**^	***p***-value non-miss.^**3**^
	N	%	N	%	N	%	N	%	N	%	N	%		
**A All cases**^**1**^	21,802	100.0	4,898	22.5	5,335	24.5	4,979	22.8	4,048	18.6	2,542	11.7		
**Vital status**														
***Alive***	13,594	62.4	3,252	66.4	3,481	65.2	3,121	62.7	2,336	57.7	1,404	55.2	<0.001	
***Dead***	8,208	37.6	1,646	33.6	1,854	34.8	1,858	37.3	1,712	42.3	1,138	44.8		
**Age at diagnosis**														
***Mean (sd)***	63.2	(14.6)	61.8	(13.9)	63.3	(14.5)	63.4	(14.7)	64.4	(15.0)	63.3	(15.2)	<0.001	
***15-49***	4,367	20.0	1,056	21.6	1,020	19.1	970	19.5	756	18.7	565	22.2	<0.001	
***50-59***	5,072	23.3	1,228	25.1	1,283	24.0	1,155	23.2	893	22.1	513	20.2		
***60-69***	5,198	23.8	1,212	24.7	1,309	24.5	1,195	24.0	913	22.6	569	22.4		
***70-79***	3,885	17.8	833	17.0	905	17.0	891	17.9	766	18.9	490	19.3		
***80+***	3,280	15.0	569	11.6	818	15.3	768	15.4	720	17.8	405	15.9		
**Period of diagnosis**														
***1989-1992***	447	2.1	93	1.9	112	2.1	115	2.3	80	2.0	47	1.8	<0.001	
***1993-1995***	1,070	4.9	284	5.8	230	4.3	248	5.0	201	5.0	107	4.2		
***1996-1998***	1,595	7.3	370	7.6	393	7.4	339	6.8	287	7.1	206	8.1		
***1999-2001***	2,585	11.9	639	13.0	616	11.5	569	11.4	475	11.7	286	11.3		
***2002-2004***	3,605	16.5	904	18.5	798	15.0	792	15.9	688	17.0	423	16.6		
***2005-2007***	5,870	26.9	1,371	28.0	1,259	23.6	1,439	28.9	1,097	27.1	704	27.7		
***2008-2010***	6,630	30.4	1,237	25.3	1,927	36.1	1,477	29.7	1,220	30.1	769	30.3		
**Stage at diagnosis**														
***Stage I***	4,912	22.5	1,116	22.8	1,286	24.1	1,137	22.8	878	21.7	495	19.5	<0.001	<0.01
***Stage II***	6,525	29.9	1,460	29.8	1,577	29.6	1,545	31.0	1,175	29.0	768	30.2		
***Stage III***	1,046	4.8	203	4.1	264	4.9	245	4.9	212	5.2	122	4.8		
***Stage IV***	516	2.4	99	2.0	127	2.4	120	2.4	98	2.4	72	2.8		
***Missing***	8,803	40.4	2,020	41.2	2,081	39.0	1,932	38.8	1,685	41.6	1,085	42.7		
**Smoking status**														
***Non-smoker***	9,464	43.4	2,362	48.2	2,442	45.8	2,165	43.5	1,625	40.1	870	34.2	0.077	<0.001
***Current smoker***	4,491	20.6	875	17.9	950	17.8	999	20.1	942	23.3	725	28.5		
***Ex-smoker***	6,705	30.8	1,449	29.6	1,711	32.1	1,539	30.9	1,227	30.3	779	30.6		
***Current or Ex-smoker***	384	1.8	52	1.1	64	1.2	92	1.8	101	2.5	75	3.0		
***Missing***	758	3.5	160	3.3	168	3.1	184	3.7	153	3.8	93	3.7		
**Alcohol drinking status**														
***Non-drinker***	3,178	14.6	555	11.3	649	12.2	721	14.5	693	17.1	560	22.0	<0.001	<0.001
***Current drinker***	12,294	56.4	2,841	58.0	3,184	59.7	2,803	56.3	2,195	54.2	1,271	50.0		
***Ex-drinker***	1,420	6.5	234	4.8	302	5.7	304	6.1	332	8.2	248	9.8		
***Missing***	4,910	22.5	1,268	25.9	1,200	22.5	1,151	23.1	828	20.5	463	18.2		
**A All cases**^**1**^	21,802	100.0	4,898	22.5	5,335	24.5	4,979	22.8	4,048	18.6	2,542	11.7		
**Body mass index (kg/m**^**2**^**)**														
***<20***	1,188	5.4	275	5.6	301	5.6	249	5.0	214	5.3	149	5.9	0.290	<0.001
***20-24***	7,195	33.0	1,903	38.9	1,828	34.3	1,565	31.4	1,210	29.9	689	27.1		
***25-29***	6,455	29.6	1,390	28.4	1,611	30.2	1,528	30.7	1,171	28.9	755	29.7		
***30+***	4,542	20.8	812	16.6	978	18.3	1,081	21.7	986	24.4	685	26.9		
***Missing***	2,422	11.1	518	10.6	617	11.6	556	11.2	467	11.5	264	10.4		
**Charlson Comorbidity Score (CPRD data only)**														
***None recorded***	16,662	76.4	4,024	82.2	4,133	77.5	3,758	75.5	2,949	72.9	1,798	70.7	<0.001	
***1***	3,597	16.5	640	13.1	842	15.8	869	17.5	741	18.3	505	19.9		
***2***	1,033	4.7	162	3.3	251	4.7	227	4.6	229	5.7	164	6.5		
***3+***	510	2.3	72	1.5	109	2.0	125	2.5	129	3.2	75	3.0		
**Consultations prior to diagnosis**														
***None recorded***	1,070	4.9	199	4.1	247	4.6	262	5.3	231	5.7	131	5.2	<0.001	
***1 to 5***	8,076	37.0	1,939	39.6	2,007	37.6	1,862	37.4	1,428	35.3	840	33.0		
***6 to 10***	6,131	28.1	1,377	28.1	1,518	28.5	1,402	28.2	1,117	27.6	717	28.2		
***11+***	6,525	29.9	1,383	28.2	1,563	29.3	1,453	29.2	1,272	31.4	854	33.6		
***Mean (sd)***	8.8	(8.3)	8.5	(7.8)	8.6	(8.0)	8.6	(8.1)	9.2	(8.8)	9.6	(9.1)		
**Breast-related consultations prior to diagnosis**														
***None recorded***	11,214	51.4	2,383	48.7	2,721	51.0	2,628	52.8	2,186	54.0	1,296	51.0	<0.001	
***1***	8,939	41.0	2,100	42.9	2,186	41.0	2,008	40.3	1,577	39.0	1,068	42.0		
***2+***	1,649	7.6	415	8.5	428	8.0	343	6.9	285	7.0	178	7.0		
***Mean (sd)***	0.58	(0.72)	0.62	(0.71)	0.60	(0.76)	0.56	(0.69)	0.55	(0.72)	0.58	(0.68)		
**Number of breast symptoms reported prior to diagnosis**														
***None recorded***	10,406	47.7	2,298	46.9	2,542	47.6	2,428	48.8	1,967	48.6	1,171	46.1	<0.01	
***1***	9,664	44.3	2,182	44.5	2,356	44.2	2,218	44.5	1,759	43.5	1,149	45.2		
***2***	1,343	6.2	328	6.7	332	6.2	273	5.5	244	6.0	166	6.5		
***3+***	389	1.8	90	1.8	105	2.0	60	1.2	78	1.9	56	2.2		
**Number of days since last symptom report to diagnosis**^**4**^														
***Mean (range)***	32.2	(1-547)	32.7	(1-541)	32.7	(1-547)	32.4	(1-545)	30.7	(1-541)	32.2	(1-541)	<0.05	
**Referrals prior to diagnosis**														
***None recorded***	14,257	65.4	3,073	62.7	3,539	66.3	3,282	65.9	2,703	66.8	1,660	65.3	<0.001	
***1***	7,044	32.3	1,691	34.5	1,667	31.2	1,590	31.9	1,261	31.2	835	32.8		
***2+***	501	2.3	134	2.7	129	2.4	107	2.1	84	2.1	47	1.8		
**B Cases diagnosed 2005-2010**^**1**^	12,500	100.0	2,608	20.9	3,186	25.5	2,916	23.3	2,317	18.5	1,473	11.8		
**Charlson comorbidity score (HES data only)**														
***None recorded***	11,774	94.2	2,512	96.3	3,032	95.2	2,742	94.0	2,137	92.2	1,351	91.7	<0.001	
***1***	451	3.6	64	2.5	101	3.2	107	3.7	110	4.7	69	4.7		
***2***	174	1.4	18	0.7	31	1.0	50	1.7	41	1.8	34	2.3		
***3+***	101	0.8	14	0.5	22	0.7	17	0.6	29	1.3	19	1.3		
**Charlson comorbidity score (CPRD or HES data)**														
***None recorded***	8,931	71.4	2,059	78.9	2,332	73.2	2,042	70.0	1,548	66.8	950	64.5	<0.001	
***1***	2,185	17.5	353	13.5	534	16.8	541	18.6	455	19.6	302	20.5		
***2***	865	6.9	124	4.8	206	6.5	211	7.2	183	7.9	141	9.6		
***3+***	519	4.2	72	2.8	114	3.6	122	4.2	131	5.7	80	5.4		
**Time from diagnosis to first major surgery**														
***On or before diagnosis***	919	7.4	176	6.7	202	6.3	206	7.1	169	7.3	166	11.3	0.064	0.106
***<=1 month***	4,583	36.7	936	35.9	1,139	35.8	1,079	37.0	888	38.3	541	36.7		
***<=2 months***	2,733	21.9	574	22.0	727	22.8	645	22.1	504	21.8	283	19.2		
***>2 months***	588	4.7	114	4.4	153	4.8	141	4.8	115	5.0	65	4.4		
***None recorded***	3,677	29.4	808	31.0	965	30.3	845	29.0	641	27.7	418	28.4		
**Number of days from diagnosis to first major surgery**^**5**^														
***Mean (sd)***	34.3	(28.4)	34.1	(27.8)	35.0	(28.9)	34.7	(29.8)	33.7	(27.4)	33.6	(27.3)	0.051	

^1^Row percentages

^2^*P*-value for association between index variable and deprivation (multinomial regression for categorical variables and non-parametric tests for trend for continuous variables, see text)

^3^*P*-value for association between index variable and deprivation, excluding missing category (multinomial regression for categorical variables and non-parametric tests for trend for continuous variables, see text)

^4^Amongst the 6,302 patients who reported a breast symptom prior to diagnosis

^5^Amongst the 7,904 patients who had surgery after diagnosis

Using information from the HES database in order to calculate the Charlson co-morbidity score for women diagnosed after 2005 did not add much: 76.4% of the cohort were identified as having no comorbidities without HES data in comparison to 71.4% with (Table 1). The distribution of co-morbidities overall was similar with 17.5% having one significant co-morbidity. A similarly strong association with deprivation was also evident (*p*-values both < 0.001). Major breast surgery was identified in 71.6% of the women in the cohort. More deprived women tended to have surgery slightly sooner overall (2.5 days earlier, 95% CI 0.4–4.7), and were more likely to have surgery at the time of or before diagnosis (11.3% in the most deprived vs 6.7% in the least, difference 4.5, 95% CI 6.4–2.6).

### Univariable survival analyses

**Fig. 2:**
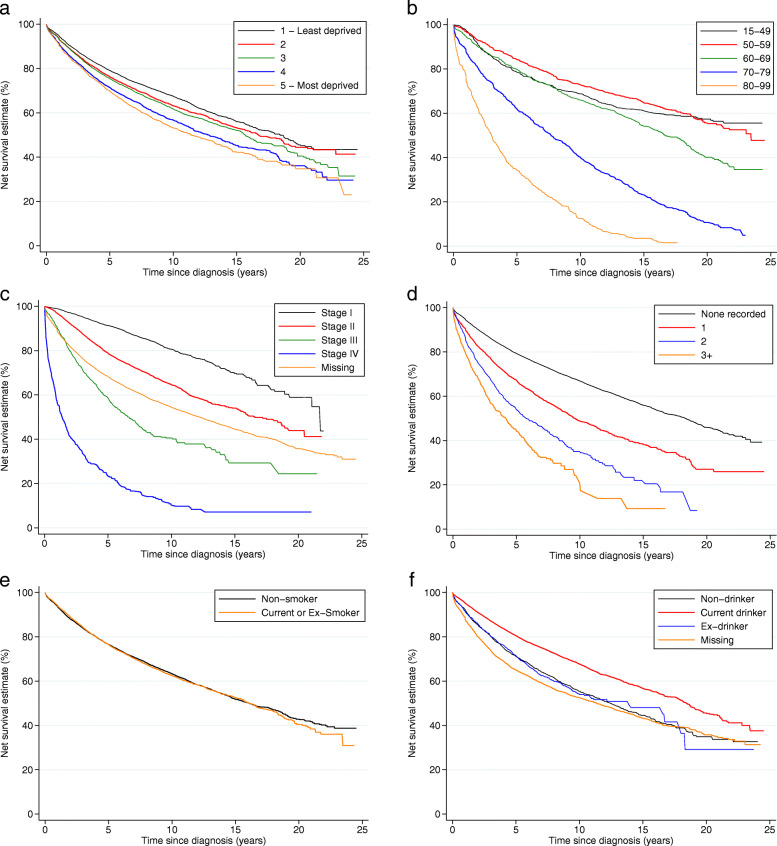
Net survival by patient demographics and individual health status: women diagnosed with breast cancer 1988–2010 and followed up to 31 December 2014

**Fig. 3:**
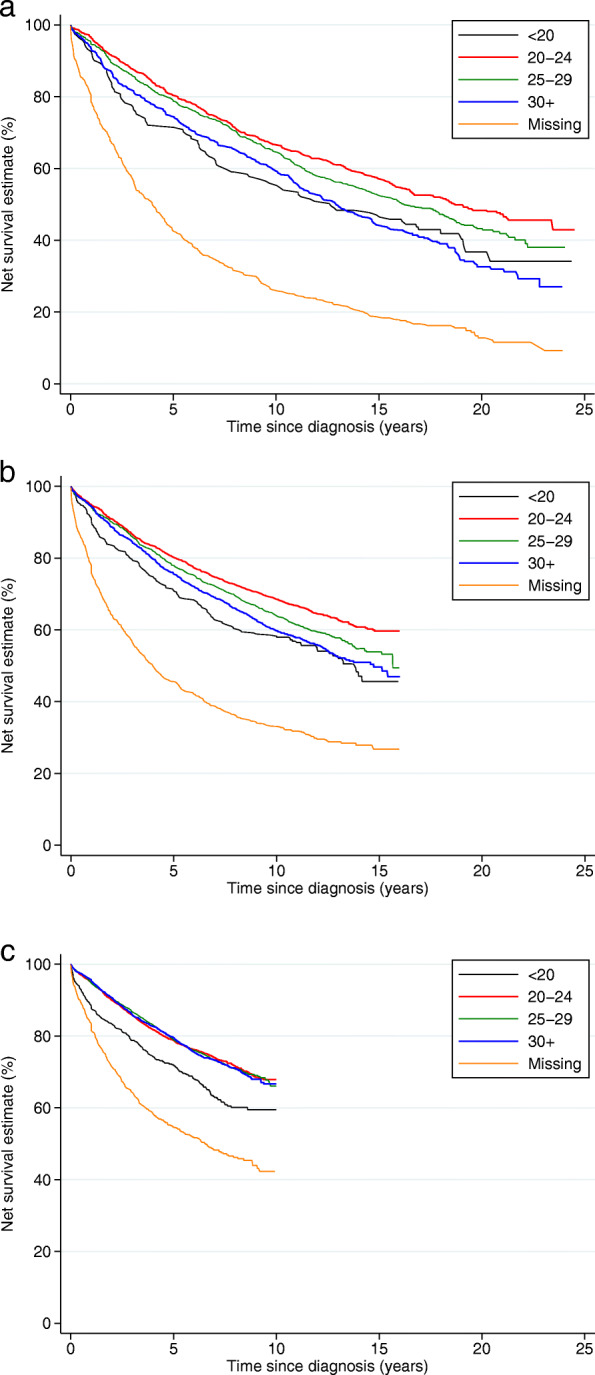
Net survival by body mass index (BMI) and period of diagnosis: women diagnosed with breast cancer 1988–2010 and followed up to 31 December 2014

**Fig. 4:**
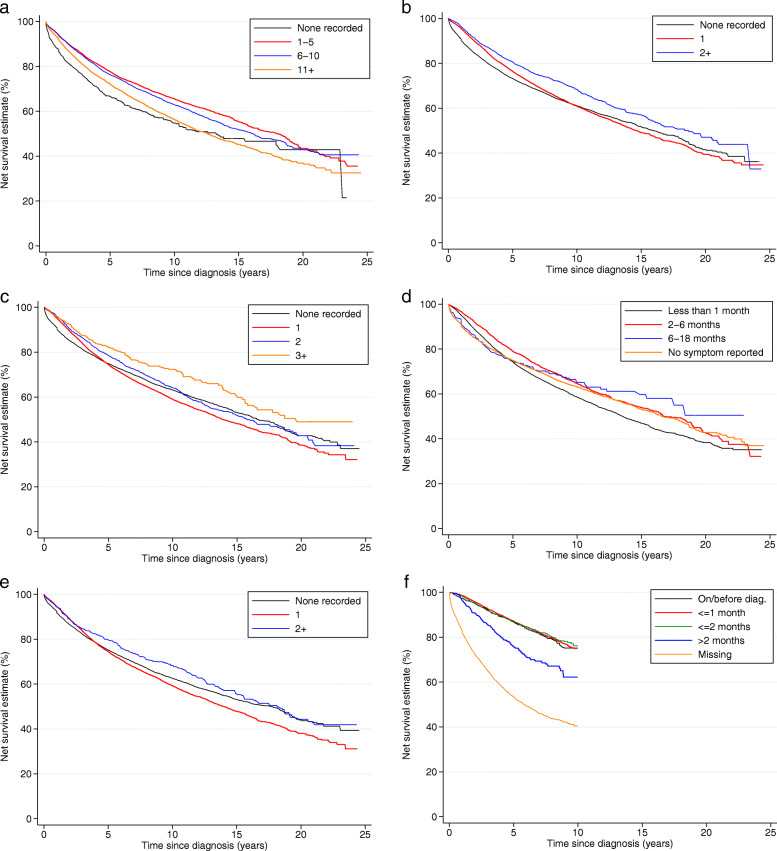
Net survival by consultation history in the 18 months prior to diagnosis: women diagnosed with breast cancer 1988–2010 and followed up to 31 December 2014

Five-year net survival increased from 71.4% (95% CI 69.8–73.0) to 76.6% (95% CI 75.9–77.4) over study period. Women living in more deprived localities had lower survival, the difference between the least and most deprived in survival (the survival ‘gap’) equal to 9% 5 years after diagnosis and 14% 10 years after diagnosis for women diagnosed during the period 2005–2010 (the post-screening era, Fig. 2a). Older women and those diagnosed at later stages displayed substantially poorer outcomes (Fig. 2b, c). Smoking status was not associated with net survival (Fig. 2e) and thus not included in the multivariable modelling. Current drinkers had better survival than non- or ex- drinkers whereas those with greater numbers of comorbidities had increasingly worse survival (Fig. 2f, d). Underweight and obese women diagnosed up to 2004 had poorer outcomes compared to those who were normal or overweight, but in the period 2005–2010 only underweight women experienced lower net survival (Fig. 3). Those with either no consultations, or more than 11 for any reason in the 18 months prior to diagnosis had worse outcomes than those who had between 1 and 10 visits to the GP, as did those who had fewer than two breast-related consultations, those whose time from last symptom report to diagnosis was shorter, and those whom received a single or no referral (Fig. 4a-e). Among women diagnosed after 2005, survival was similar irrespective of time from diagnosis to breast surgery, except amongst women whose time to surgery was greater than 2 months or surgical status was missing amongst whom survival was dramatically worse (Fig. 4f).

### Multivariable excess hazard modelling

**Fig. 5:**
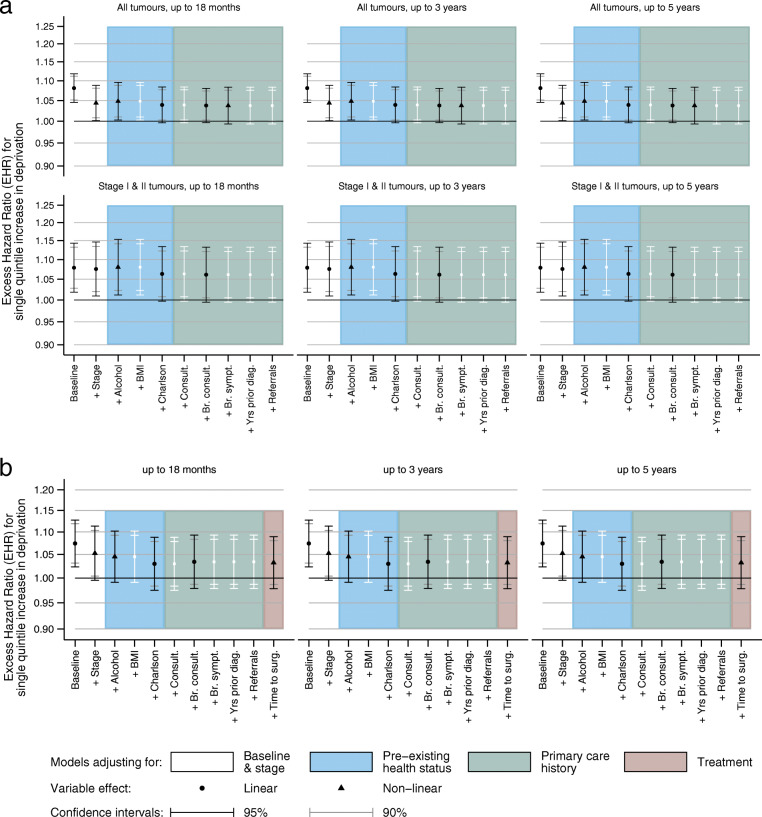

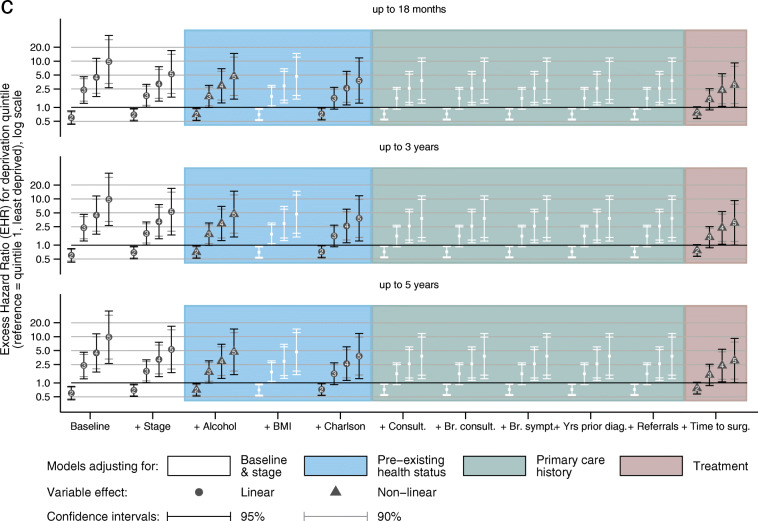
Excess hazard ratio of breast cancer death associated with increasing deprivation^a^: baseline and adjusted^b^ in multivariable models fitted to imputed data. **a**) Women diagnosed 1 January 1988–31 December 2010 followed up to 31 December 2014, all tumours and early stages. **b**) Women diagnosed 1 January 2005–31 December 2010 followed up to 31 December 2014, all tumours. **c**) Women diagnosed 1 January 2005–31 December 2010 followed up to 31 December 2014, early stages. ^a^ Single unit increases derived from linear models are displayed with solid symbols. Where the effect was found to be non-linear, (e.g. (c)) numbers displayed within the symbols correspond to the deprivation quintile compared to the least deprived group (quintile 1). ^b^ Symbols are displayed *only* when the addition of the variable resulted in a significant improvement in the model fit (*p* < 0.05). Variable descriptions (see text for full coding), *Baseline:* Model adjusted for age and year of diagnosis only, *Stage:* Stage of disease at diagnosis, *Alcohol:* Drinking habits, *BMI:* Body Mass Index (kg/m^2^), *Charlson:* Charlson co-morbidity score, *Consult.:* Number of consultations for any reason, *Br. Consult.:* Number of consultations for breast symptom, *Br. Sympt:* Number of breast symptoms reported, *Yrs prior diag:* Number of days from last breast-related consultation to diagnosis, *Referrals:* Number of referrals for breast cancer, *Time surg.*: Number of days from diagnosis to major breast surgery

After accounting for age, year and stage at diagnosis, a single unit increase in deprivation quintile was associated with a significant 4.4% (95% CI 1.4–8.8) increase in excess mortality due to breast cancer across all periods of follow-up time (Fig. 5a) in the imputed data. Amongst women diagnosed with stage I or II disease, the differential was greater (7.6, 95% CI 0.9–14.6) but of borderline significance. These hazard ratios equate to a 17.5% (or 30.3% for stages I & II) mortality differential between the most affluent and most deprived groups. A similarly consistent linear association was observed amongst women diagnosed 2005–2010 (Fig. 5b) and for all the different age groups (data not shown).

The inclusion of co-variables relating to individual health status, primary and secondary care had almost no impact on the magnitude of the differential amongst those diagnosed 1988–2010 and minimal impact for those diagnosed 2005–2010. Significant variables in the multivariable models were restricted to alcohol intake, comorbidity and the number of breast consultations. The number of breast symptoms reported was significant for all women across the study period but not for those diagnosed with early stage disease nor those diagnosed after 2004. Time to breast surgery (available only for women diagnosed after 2004) significantly improved the fit but did not alter the magnitude of the association.

Women diagnosed with early stage disease between 2005 and 2010 who were living in areas categorised as quintile 2 had lower excess mortality than women living in quintiles 1, 3, 4 or 5 (Fig. 5c). Similar to the above, only alcohol consumption and comorbidity improved fit of these non-linear stage-adjusted models, but the number of consultations did not. Time to breast surgery improved the model fit but reduced the magnitude of the associations slightly.

## Discussion

### Summary

We have shown that individual health status at diagnosis and primary care consultation history vary by deprivation status but do not explain socio-economic differences in breast cancer survival in this cohort as far as can be established from these data. A persistent and consistent increase in deprivation-specific cancer mortality was observed. Although the association did not reach significance for women diagnosed most recently, its magnitude was almost identical to that for the period as a whole. The accuracy and completeness of some fields utilised in this study could be improved. Nevertheless these data support the null hypothesis that socio-economic differentials in breast cancer survival are not primarily explained by pre-existing individual health status and primary care consultation history.

### Strengths and limitations

We used a unique, national, population-based, individually-linked database. This included three separate measures of individual health status, a single measure of pre-existing comorbidities and pre-diagnostic consultation rate both overall and for breast complaints specifically. We used the most up-to-date survival analysis methodology [32] combined with deprivation-specific estimates of background mortality, and have simultaneously examined the impact of multiple peri-diagnostic factors upon the excess hazard due to the disease [35].

We defined a woman’s deprivation category based upon the characteristics of her local area. Consequently, we have demonstrated the influence of ecologically-measured deprivation, rather than of individual circumstances. Although LSOAs are designed to be as socially homogenous as possible, it is probable that more deprived individuals are distributed across the different quintiles of ecological deprivation. Since personal socio-economic data are not available in either the CPRD or cancer registration databases evaluating the direct impact of individual deprivation is not possible in these data. The differentials we identify will are thus likely to reflect the impact of both environmental (contextual) and individual deprivation. The extent to which each are independently influential remains to be demonstrated.

Our database included a substantial proportion of missing data, most importantly for stage, alcohol consumption, and BMI. We accounted for these by multiple imputation methods. Although we examined the likely mechanisms giving rise to missing data, some residual bias may still be present. For comorbidity, consultations, referrals and symptoms there was no missing data simply because it was not possible to distinguish between, for example, a patient with no pre-existing comorbidities and one with unrecorded comorbidities. Further, our measures of BMI, smoking and alcohol only capture a part of the differences in underlying health status, nutrition and physical activity. Residual confounding is thus likely to be present. Our analysis of number of symptoms, referrals, comorbidities, and time to major breast surgery assumed that ‘none recorded’ equated to ‘none observed’. This is a limitation as some of these groupings are likely to, in fact, represent persons who did report symptoms, were referred or received surgery but for whom this information is missing. In particular, it has been noted that affluent women are more likely to undergo surgery in the private sector, which is undetectable in the HES database [11]. We did not have very detailed information on surgery (mastectomy, breast conserving therapy) or other types of treatment received (radiotherapy, chemotherapy, hormonal treatments) which may potentially explain some of the differences observed and could be included in further analyses for periods in which these fields are more complete. For example, the effectiveness of the surgery (experience of surgeon, hospital, and neo-adjuvant therapies given) may vary with deprivation. Finally, we were unable to define women by ethnicity in these analyses. Black women are known to have lower breast survival than White or South Asian women [9], in part due to more aggressive tumours. We were unable to account for this but it is unlikely to substantially bias our results since Black women are a very small proportion (< 3%) of the overall population [37].

### Comparison with existing literature

These data are consistent with those we previously reported which showed that neither individual health status nor primary care consultation patterns explain much of survival inequalities amongst women diagnosed in the screening age range [20], as well as a notable ‘J’ shaped relationship between deprivation and survival for women with early stage disease [38]. The data we present here on stage I & II disease are also consistent with our demonstration that socio-economic differentials in net survival are present amongst women whose tumour was screen-detected [10].

More deprived women in our study were no less likely to consult their GP, in fact, they consulted slightly more and reported a similar number of symptoms. This may seem counter-intuitive given their more advanced disease at diagnosis and lower survival. However, it is consistent with other data from the UK [39, 40], Denmark [41] and Australia [42], as well as with an ecological study of healthcare trusts in England which showed symptom awareness for breast cancer was similar across the socio-economic spectrum, although help-seeking behaviours were slightly lower in more deprived areas [43]. Breast cancer is characterised by especially short pre-diagnosis presentation intervals [44] which may suggest that the lack of association observed here between peri-diagnostic factors and survival is unique to this malignancy. However, peri-diagnostic consultation rates have also been shown to be similar amongst colon cancer patients presenting as emergencies compared to non-emergencies [45]. Since emergency presentation is much more frequent amongst more deprived patients [46] this lends weight to the interpretation that the lower cancer survival experienced by more deprived cancer patients in general are not primarily related to differential use or access to primary care.

### Implications for future research

This study has shown that the underlying reasons for socio-economic differentials in cancer survival are elusive but are not likely to fall exclusively in the peri-diagnostic period. It is known that more deprived women are disproportionately diagnosed with the most aggressive, triple negative tumours which may partially explain these observations [47, 48]. The fact that a greater number of deprived women had major surgery at the time of or prior to diagnosis may further suggest that they are more frequently diagnosed via the emergency route or opportunistically, but this is known to be rare for breast cancer. Beyond this, timing of surgery did not strongly influence survival except where it was > 2 months or missing, and was not strongly socio-economically patterned (Table 1). However, it remains the case that variations in treatment effectiveness, beyond the inclusion of major breast surgery and the timing of surgery [21], may have a significant role in determining differentials in outcomes. Future investigations might examine differences in the types of hospital patients to travel to [49], differential experience and resources available in different centres [50], as well as the types of treatment and follow-up patients are offered, or opt to receive [51], and the timing of each of these events.

## Conclusions

We have demonstrated that socio-economic inequalities in survival in these data cannot be explained by consultation history or by pre-existing individual health status, as measured in primary care. The absolute impact of the differentials demonstrated here is relatively small for women with breast cancer since the excess mortality rate itself is now, mercifully, fairly low. However, it is probable that these patterns are suggestive of a tendency towards differential treatment effectiveness which has wide ranging implications for cancers or other diseases with socio-economically patterned outcomes where treatment effectiveness is likely to be similarly differentiated. Since reducing inequalities in premature mortality is a major focus of current health policy in England [52], effort should be made to develop a better understanding of the causes and perpetuation of socio-economic health differentials in secondary as well as primary care.

## Data Availability

The data used in this article are not publically available but can be accessed via CPRD: www.cprd.com

## Abbreviations

CPRD: Clinical Practice Research Datalink
HES: Hospital Episodes Statistics
CR: English National Cancer Registry
IMD: Indices of Multiple Deprivation
ED: Enumeration District
LSOA: Lower-level Super Output Area
GP: General Practitioner
OPCS-4: Office of Population Censuses and Surveys Classification of Interventions and Procedures version 4
BMI: Body Mass Index (kg/m^2^)
TNM: Tumour, Nodes and Metastases Classification of Malignant Tumours
